# Re-evaluating the case for poecilogony in the gastropod *Planaxis sulcatus* (Cerithioidea, Planaxidae)

**DOI:** 10.1186/s12862-022-01961-7

**Published:** 2022-02-07

**Authors:** Giulia Fassio, Philippe Bouchet, Marco Oliverio, Ellen E. Strong

**Affiliations:** 1 Department of Biology and Biotechnologies “Charles Darwin”, Sapienza University of Rome, Zoology–Viale dell’Università 32, 00185 Rome, Italy; 2 Department of Biology and Evolution of Marine Organisms, Stazione Zoologica Anton Dohrn, Villa Comunale, 80121 Naples, Italy; 3 Institut de Systématique, Évolution, Biodiversité ISYEB–UMR 7205–CNRS, MNHN, UPMC, EPHE, Muséum National d’Histoire Naturelle, Sorbonne Université, Paris, France; 4 Department of Invertebrate Zoology, National Museum of Natural History, Smithsonian Institution, Washington, DC 20013 USA

**Keywords:** Reproductive biology, Cryptic species, Larval development, Viviparity

## Abstract

**Background:**

*Planaxis sulcatus* has been touted as a textbook example of poecilogony, with members of this wide-ranging Indo-Pacific marine gastropod said to produce free-swimming veligers as well as brooded juveniles. A recent paper by Wiggering et al. (BMC Evol Biol 20:76, 2020) assessed a mitochondrial gene phylogeny based on partial COI and 16S rRNA sequences for 31 individuals supplemented by observations from the brood pouch of 64 mostly unsequenced individuals. ABGD and bGYMC supported three reciprocally monophyletic clades, with two distributed in the Indo-Pacific, and one restricted to the northern Indian Ocean and Red Sea. Given an apparent lack of correlation between clade membership and morphological differentiation or mode of development, the reported 3.08% maximum K2P model-corrected genetic divergence in COI among all specimens was concluded to represent population structuring. Hence, the hypothesis that phylogenetic structure is evidence of cryptic species was rejected and *P. sulcatus* was concluded to represent a case of geographic poecilogony.

**Results:**

Our goal was to reassess the case for poecilogony in *Planaxis sulcatus* with a larger molecular dataset and expanded geographic coverage. We sequenced an additional 55 individuals and included published and unpublished sequence data from other sources, including from Wiggering et al. Our dataset comprised 108 individuals (88 COI, 81 16S rRNA) and included nine countries unrepresented in the previous study. The expanded molecular dataset yielded a maximum K2P model-corrected genetic divergence among all sequenced specimens of 12.09%. The value of 3.08% erroneously reported by Wiggering et al. is the prior maximal distance value that yields a single-species partition in ABGD, and not the maximum K2P intraspecific divergence that can be calculated for the dataset. The bGMYC analysis recognized between two and six subdivisions, while the best-scoring ASAP partitions recognized two, four, or five subdivisions, not all of which were robustly supported in Bayesian and maximum likelihood phylogenetic analyses of the concatenated and single gene datasets. These hypotheses yielded maximum intra-clade genetic distances in COI of 2.56–6.19%, which are more consistent with hypothesized species-level thresholds for marine caenogastropods.

**Conclusions:**

Based on our analyses of a more comprehensive dataset, we conclude that the evidence marshalled by Wiggering et al. in support of *Planaxis sulcatus* comprising a single widespread, highly variable species with geographic poecilogony is unconvincing and requires further investigation in an integrative taxonomic framework.

**Supplementary Information:**

The online version contains supplementary material available at 10.1186/s12862-022-01961-7.

## Background

Marine invertebrates can undergo one of two types of larval development [1]. In the planktotrophic type of development, a free-swimming planktonic larva feeds on phytoplankton, typically during a period of several weeks or months, or occasionally years; in the non-planktotrophic (also called lecithotrophic or, sometimes incorrectly, direct) type of development, the larva reaches metamorphosis without phytoplanktonic food uptake [2] and typically spends a few days or less in the water column. The mode of larval development is a species attribute that is usually difficult to determine as, in most phyla, it requires collecting eggs from spawning adults and raising the larvae. In shelled Gastropoda, however, as a consequence of the accretionary growth of the shell, the protoconch—or larval shell, very frequently retained at the apex of the adult shell—reflects the mode of development; a so-called multispiral protoconch is indicative of planktotrophic larval development, and a so-called paucispiral protoconch is indicative of non-planktotrophic larval development [3]. This extraordinary correlation can be traced in the fossil record [4], and gastropod protoconchs can be attributed to either a planktotrophic or a non-planktotrophic mode of development already in Palaeozoic fossils. Not surprisingly, the protoconch has been used as a species-specific character, with many examples of species pairs diverging in inferred development type, and developmental shifts suggested to represent a major driver of speciation (see e.g. [5]).

The literature contains reports of rare cases when larvae with the two modes of development are produced by the same individual, population, or species [6, 7], a phenomenon termed poecilogony. Notably, even among the rare and established cases of poecilogony, there is still only a single species (*Alderia willowi* Krug, Ellingson, Burton & Valdés, 2007) in which one individual has been shown to vary the development mode of its offspring [8]. However, if the phenomenon were widespread, this would of course severely weaken the value of the multispiral/paucispiral protoconch dichotomy as a taxonomic character. Whereas poecilogony has been confirmed in several species of sea-slugs [9–11]—and, outside molluscs, in polychaetes [12]—, alleged cases of poecilogony in shelled gastropods were reviewed in pre-molecular times by Hoagland and Robertson [13] and Bouchet [14] who independently concluded that there were no definitive cases of poecilogony in marine shelled gastropods. However, McDonald et al. [15] and Russini et al. [16] did provide molecular data suggesting the existence of very rare cases of poecilogony in Caenogastropoda, although these represented different taxa from the cases traditionally reported in the historical literature.

One such historically reported case concerns the marine snail *Planaxis sulcatus* (Born, 1778), a common intertidal gastropod with a broad Indo-Pacific distribution, from the East African coasts of the Indian Ocean and the satellite Red Sea and Persian Gulf, to the western central Pacific. Risbec [17] and Thorson [18] reported contradictory observations on its mode of development in New Caledonia and the Persian Gulf, respectively. In New Caledonia, Risbec described a classical planktotrophic larval development, whereas in the Persian Gulf the same species was reported to incubate the young, which feed on nurse eggs and metamorphose before hatching. Although Bouchet [12] hypothesized the existence of two cryptic species, each with its own species-specific mode of development, the *Planaxis sulcatus* case has been highlighted by Wiggering et al. [19] as a “textbook example for poecilogony”.

Planaxidae constitute a small family of tropical/temperate cerithioidean gastropods including about 60 Recent species. They are all restricted to the upper/middle intertidal, where they may form large aggregations, either on/under stones (Planaxinae) or in crevices (Fossarinae), where they presumably graze on the biofilm. Many species brood their fertilized eggs in a subhaemocoelic brood pouch located in the neck region of the headfoot, until larvae or juveniles are released.

Wiggering et al. [19] observed that different populations of *Planaxis sulcatus* exhibit different modes of larval development, with Western Indian Ocean and Red Sea populations releasing large, shelled juveniles from the brood pouch, whereas the Indo-West Pacific populations release planktotrophic veliger larvae. They sequenced 31 specimens of *P. sulcatus* from throughout the geographical range and concluded that their data confirmed *P. sulcatus* as a single species rather than a group of cryptic species, thus representing a case of geographic poecilogony.

If poecilogony occurs in *Planaxis sulcatus*, and other caenogastropods, then the paradigm of protoconch dichotomy (multispiral v. paucispiral) as a species-specific character would be severely weakened, with profound consequences for the alpha-taxonomy of extant and fossil molluscs. The genetic mechanisms involved in larval ecology of marine invertebrates (including intraspecific variation) are still largely unknown, and poecilogonous taxa would be the best models for such studies [20]. However, the acceptance of this extraordinary claim requires that the evidence and its interpretation are unambiguous: for poecilogony to be robustly inferred, the two modes of larval development must be found to occur—or at least be reliably inferred to occur—among the offspring of the same individual, or among individuals of the same or different populations of a single species.

The objective of the present work is thus to review the case for alleged poecilogony in *Planaxis sulcatus* with an expanded molecular dataset, and to test the alternative hypothesis that *P. sulcatus* in fact comprises a complex of cryptic species potentially differing in development.

## Methods

### Material

**Fig. 1.**
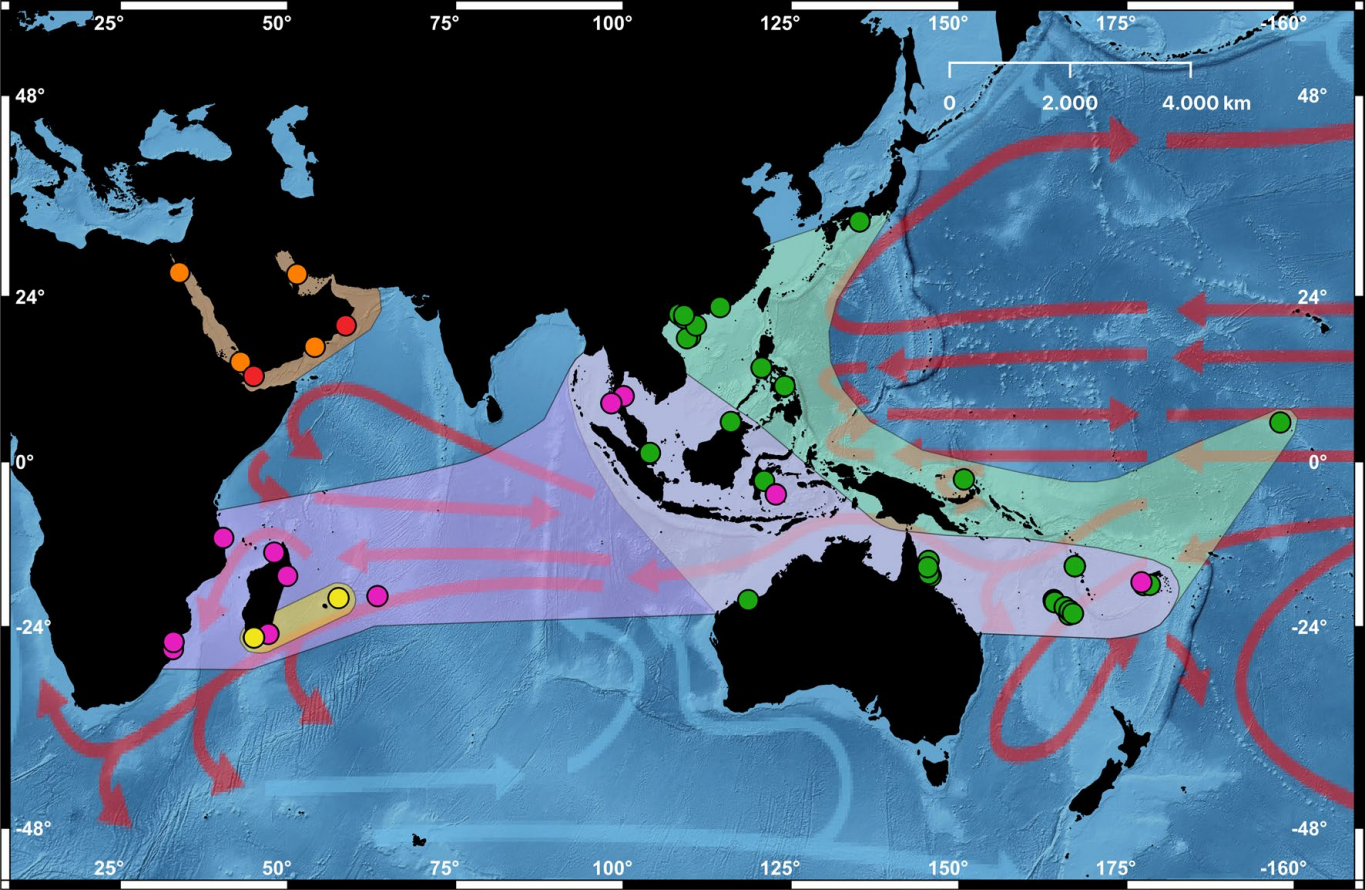
Distribution of *Planaxis sulcatus* sequenced specimens. Green, Clade I; yellow, Clade IIa; red, Clade IIb; orange, Clade IIc; purple, Clade III. Arrows indicate warm (red) and cold (blue) surface ocean currents (source: US National Oceanic and Atmospheric Administration)

Wiggering et al. [19] generated a dataset of partial COI and 16S rRNA mitochondrial gene sequences from 31 museum specimens. Owing to difficulties in extracting and amplifying DNA from historical material, they were successful in obtaining only 16 COI and 28 16S rRNA sequences. We were able to augment this from published and unpublished sources, and with newly generated sequences from recently collected specimens in the collections of the Muséum national d’Histoire naturelle in Paris (MNHN), National Museum of Natural History in Washington, DC (USNM), and Florida Museum of Natural History (UF). Our dataset comprises 108 specimens of *Planaxis sulcatus* from three biogeographic realms (Western Indo-Pacific, Central Indo-Pacific, and Temperate Northern Pacific), plus one specimen collected in the Eastern Indo-Pacific (Palmyra Atoll) (following [21]; Fig. 1). Sequences for 53 specimens were retrieved from GenBank and BOLD, including those published by Wiggering et al. [19], and unpublished sequences for seven individuals were kindly shared with us by colleagues. Sequences for the two outgroups used by Wiggering et al. [19], *Supplanaxis niger* and *Planaxis planicostatus*, and for *Planaxis* sp. were also downloaded from GenBank. See Table 1 for details.

**Table 1:** Registration numbers, GenBank/BOLD accession numbers, and collecting sites of sequenced specimens

Clade	Species	Voucher	Collection data	Source	COI	16S
I	*Planaxis sulcatus*	AMS 322974-h2	New Caledonia, Ilot Maitre, in channel near Nouméa, 22° 20'S, 166° 24'E	[19]	–	MT621367
I	*Planaxis sulcatus*		Australia, Queensland, Cairns	[22]	–	AY010320
I	*Planaxis sulcatus*	LSGB M 014	China, Qingshuiwan, Lingshui, Hainan Island	[23]	MN389032	–
I	*Planaxis sulcatus*	LSGB21101	China, Beihai, Guangxi Province, 21° 26' N, 109° 04' E	[24]	–	HQ833974
I	*Planaxis sulcatus*	MDV108	Japan, Gobo, Wakayama Prefecture	[25]	LC415042	–
I	*Planaxis sulcatus*	MDV109	Japan, Gobo, Wakayama Prefecture	[25]	LC415043	–
I	*Planaxis sulcatus*	MDV111	Japan, Gobo, Wakayama Prefecture	[25]	LC415044	–
I	*Planaxis sulcatus*	MNHM-IM-2009-31619	Australia, Cooke Point, Port Hedland	Present work	MZ470528	MZ470478
I	*Planaxis sulcatus*	MNHN-IM-2009-31620	Australia, Cooke Point, Port Hedland,	Present work	MZ470532	MZ470482
I	*Planaxis sulcatus*	MNHN-IM-2007-32461	Vanuatu, W Aésé Island, SANTO 2006, VM32, 15° 26.6′S, 167° 15.2'E	Present work	MZ470529	MZ470479
I	*Planaxis sulcatus*	MNHN-IM-2007-32463	Vanuatu, Palikulo Peninsula, SANTO 2006, VM11, 15° 28.8′ S, 167° 15.3′ E	Present work	–	MZ470480
I	*Planaxis sulcatus*	MNHN-IM-2007-32465	Vanuatu, Palikulo Peninsula, SANTO 2006, VM11, 15° 28.8′ S, 167° 15.3′ E	Present work	MZ470530	–
I	*Planaxis sulcatus*	MNHN-IM-2007-32466	Vanuatu, W Aésé Island, SANTO 2006, VM32, 15° 26.6′ S, 167° 15.2′ E	Present work	MZ470531	MZ470481
I	*Planaxis sulcatus*	MNHN-IM-2013-42234	Singapore, Changi Coast	Present work	MZ470533	MZ470483
I	*Planaxis sulcatus*	MNHN-IM-2013-42235	Singapore, Changi Coast	Present work	MZ470534	MZ470484
I	*Planaxis sulcatus*	MNHN-IM-2013-42236	Singapore, Changi Coast	Present work	MZ470535	MZ470485
I	*Planaxis sulcatus*	MNHN-IM-2013-42237	Singapore, Changi Coast	Present work	MZ470536	MZ470486
I	*Planaxis sulcatus*	MNHN-IM-2013-42238	Singapore, Changi Coast	Present work	MZ470537	MZ470487
I	*Planaxis sulcatus*	MNHN-IM-2013-50578	Papua New Guinea, N coast of Globig [Enuk 2] I., KAVIENG 2014, KM18, 02° 38.6′ S, 150° 43.5′ E	Present work	MZ470538	MZ470488
I	*Planaxis sulcatus*	MNHN-IM-2013-50584	Papua New Guinea, N coast of Globig [Enuk 2] I., KAVIENG 2014, KM18, 02° 38.6′ S, 150° 43.5′ E	Present work	MZ470539	MZ470489
I	*Planaxis sulcatus*	MNHN-IM-2013-80052	New Caledonia, Pointe Pandop, KOUMAC 2.1, KM100, 20° 34.9′ S, 164° 16.6′ E	Present work	MZ470540	MZ470490
I	*Planaxis sulcatus*	MNHN-IM-2013-80053	New Caledonia, Pointe Pandop, KOUMAC 2.1, KM100, 20° 34.9′ S, 164° 16.6′ E	Present work	MZ470541	MZ470491
I	*Planaxis sulcatus*	MNHN-IM-2013-80054	New Caledonia, Pointe Pandop, KOUMAC 2.1, KM100, 20° 34.9′ S, 164° 16.6′ E	Present work	MZ470542	MZ470492
I	*Planaxis sulcatus*	MNHN-IM-2013-80055	New Caledonia, Pointe Pandop, KOUMAC 2.1, KM100, 20° 34.9′ S, 164° 16.6′ E	Present work	MZ470543	MZ470493
I	*Planaxis sulcatus*	MNHN-IM-2013-80056	New Caledonia, Pointe Pandop, KOUMAC 2.1, KM100, 20° 34.9′ S, 164° 16.6′ E	Present work	MZ470544	MZ470494
I	*Planaxis sulcatus*	MNHN-IM-2013-80075	New Caledonia, Ilot Rat, KOUMAC 2.1, KM200, 20° 33.4′ S, 164° 10.9′ E	Present work	MZ470545	MZ470495
I	*Planaxis sulcatus*	MNHN-IM-2013-80076	New Caledonia, Ilot Rat, KOUMAC 2.1, KM200, 20° 33.4′ S, 164° 10.9′ E	Present work	MZ470546	MZ470496
I	*Planaxis sulcatus*	MNHN-IM-2019-16088	New Caledonia, Touaourou, 22° 11.277'S, 166° 58.642'E	Present work	MZ470547	MZ470497
I	*Planaxis sulcatus*	MNHN-IM-2019-16089	New Caledonia, Touaourou, 22° 11.277'S, 166° 58.642'E	Present work	–	MZ470498
I	*Planaxis sulcatus*	MNHN-IM-2019-16090	New Caledonia, Malabou, 20° 17.510′ S, 164° 06.438′ E	Present work	MZ470548	MZ470499
I	*Planaxis sulcatus*	MNHN-IM-2019-16091	New Caledonia, Malabou, 20° 17.510′ S, 164° 06.438′ E	Present work	MZ470549	MZ470500
I	*Planaxis sulcatus*	MNHN-IM-2019-16092	New Caledonia, Cap des Trois Sapins, 21° 17.918′ S, 165° 45.265′ E	Present work	MZ470550	MZ470501
I	*Planaxis sulcatus*	MNHN-IM-2019-16093	New Caledonia, Cap des Trois Sapins, 21° 17.918′ S, 165° 45.265′ E	Present work	MZ470551	MZ470502
I	*Planaxis sulcatus*	MNHN-IM-2019-16094	New Caledonia, Cap des Trois Sapins, 21° 17.918′ S, 165° 45.265′ E	Present work	MZ470552	MZ470503
I	*Planaxis sulcatus*	MNHN-IM-2019-16095	New Caledonia, Petit Borindi, 21° 47.783′ S, 166° 29.620′ E	Present work	MZ470553	MZ470504
I	*Planaxis sulcatus*	MNHN-IM-2019-16096	New Caledonia, Petit Borindi, 21° 47.783′ S, 166° 29.620′ E	Present work	MZ470554	MZ470505
I	*Planaxis sulcatus*	MNHN-IM-2019-16097	New Caledonia, Petit Borindi, 21° 47.783′ S, 166° 29.620′ E	Present work	MZ470555	MZ470506
I	*Planaxis sulcatus*	MNHN-IM-2019-16131	Philippines, Luzon, Calantagan, Batangas, 13° 55.319′ N, 120° 37.260′ E	Present work	MZ470556	–
I	*Planaxis sulcatus*	MNHN-IM-2019-16132	Philippines, Luzon, Lian, Batangas, 13° 58.130′ N, 120° 37.471′ E	Present work	MZ470557	–
I	*Planaxis sulcatus*	MNHN-IM-2019-16133	Australia, Queensland, Cairns, Yule Point, 16° 34.226′ S, 145° 30.580′ E	Present work	MZ470558	–
I	*Planaxis sulcatus*	NSMT Mo70801	Philippines, N Cebu Island	[26]	–	FJ606927
I	*Planaxis sulcatus*	UF 295967	Fiji, Viti Levu Island, Vilisites	Present work	MZ470559	MZ470507
I	*Planaxis sulcatus*	UF 330842	Fiji, Viti Levu Island, Laucala Bay, behind USP Maritime Studies buildings, 18° 09′ 03″ S, 178° 27′ 14″ E	Present work	MZ470560	MZ470508
I	*Planaxis sulcatus*	UF 411832	USA, Line Islands, Palmyra Atoll, shore and reef margin around Sand Islet, 5° 52′ 36″ N, 162° 06′ 18″ W	Present work	MZ470561	MZ470509
I	*Planaxis sulcatus*	UF 463999	Singapore, Raffles Light House, Coney Islet	Present work	MZ470562	MZ470510
I	*Planaxis sulcatus*	USNM 1643576	Australia, off Lizard Island, South (Newt) Island, 14° 42.089′ S, 145° 27.418′ E	Present work	MZ470563	MZ470511
I	*Planaxis sulcatus*	USNM 1643577	Australia, off Lizard Island, South (Newt) Island, 14° 42.089′ S, 145° 27.418′ E	Present work	MZ470564	MZ470512
I	*Planaxis sulcatus*	USNM 1643578	Australia, off Lizard Island, South (Newt) Island, 14° 42.089′ S, 145° 27.418′ E	Present work	MZ470565	MZ470513
I	*Planaxis sulcatus*	X131	China, Weizhoudao, Guangxi Province	[27]	JF693413	–
I	*Planaxis sulcatus*	X133	China, Fangchenggang, Guangxi Province	[27]	JF693411	–
I	*Planaxis sulcatus*	X134	China, Wenchang, Hainan Province	[27]	JF693414	–
I	*Planaxis sulcatus*	X135	China, Sanya, Hainan Province	[27]	JF693412	–
I	*Planaxis sulcatus*	XN18	China, Shenzhen, Guangdong Province	[27]	JF693416	–
I	*Planaxis sulcatus*	XN33	China, Beihai, Guangxi Province	[27]	JF693415	–
I	*Planaxis sulcatus*	ZMB 106372-1	Australia, Queensland, Archer Point Rock, S Cooktown, 15° 36.250′ S, 145° 19.590′ E	[19]	–	MT621371
I	*Planaxis sulcatus*	ZMB 106372-2	Australia, Queensland, Archer Point Rock, S Cooktown, 15° 36.250′ S, 145° 19.590′ E	[19]	–	MT621372
I	*Planaxis sulcatus*	ZMB 106461-1	Indonesia, Southeast Sulawesi, Raha, fortified beach in front of Hotel ″ Alia″ , 04° 50.500′ S, 122° 43.540′ E	[19]	MT587886	MT593028
I	*Planaxis sulcatus*	ZMB 107593-4	Australia, Queensland, Yule Point, south of Port Douglas	[19]	–	MT621373
I	*Planaxis sulcatus*	ZMB 107725-4	Thailand, Gulf of Siam, E coast of Koh Phangan, Ao Thong Nai Pan Noi, 9° 46.883′ N, 100° 3.355′ E	[19]	MT620953	MT621374
I	*Planaxis sulcatus*	ZMB 107725-6	Thailand, Gulf of Siam, E coast of Koh Phangan, Ao Thong Nai Pan Noi, 9° 46.883′ N, 100° 3.355′ E	[19]	MT587885	MT593027
I	*Planaxis sulcatus*	ZMB 107933-2	Thailand, Gulf of Siam, E coast of Koh Phangan, Ao Thong Nai Pan Noi, 9° 46.883′ N, 100° 3.355′ E	[19]	MT620955	MT621376
I	*Planaxis sulcatus*	ZMB 107933-3	Thailand, Gulf of Siam, E coast of Koh Phangan, Ao Thong Nai Pan Noi, 9° 46.883′ N, 100° 3.355′ E	[19]	MT620956	MT621377
I	*Planaxis sulcatus*	ZMB 108267-3	Australia, Queensland, Cape Tribulation, Donovan Bay	[19]	–	MT621378
I	*Planaxis sulcatus*	ZMB 108275-1	Malaysia, Borneo, Sabah, Kota Kinabalu, Tanjung Aru	[19]	–	MT621379
I	*Planaxis sulcatus*	ZMB 108275-2	Malaysia, Borneo, Sabah, Kota Kinabalu, Tanjung Aru	[19]	–	MT621380
I	*Planaxis sulcatus*	ZMB 191632-9	Indonesia, Southeast Sulawesi, Peninsula S of Malili, between Malili and Tolala, just E of Cape Pagara, 2° 52.179′ S, 120° 59.915′ E	[19]	–	MT621390
IIa	*Planaxis sulcatus*	MNHN-IM-2009-27091	Madagascar, Ambatobe, Bavarama, ATIMO VATAE, BM06, 25° 27.9′ S, 44° 57.6′ E	Present work	MZ470566	MZ470514
IIa	*Planaxis sulcatus*	MNHN-IM-2009-27109	Madagascar, Ambatomainty, ATIMO VATAE, BM03, 25° 26.3′ S, 44° 56.5′ E	Present work	MZ470567	–
IIa	*Planaxis sulcatus*	MNHN-IM-2009-27143	Madagascar, Ambatomainty, ATIMO VATAE, BM03, 25° 26.3′ S, 44° 56.5′ E	Present work	MZ470568	–
IIa	*Planaxis sulcatus*	ZMB 117937-1	Mauritius, Mont Choisy	[19]	–	MT621386
IIb	*Planaxis sulcatus*	CWR 17/07-1	Yemen, Little Aden	[19]	–	MT621369
IIb	*Planaxis sulcatus*	UF 292851	Oman, Masirah Island, off camp on south-southwest coast of Masirah, 20° 00′ 40″ N, 58° 38′ 00″ E,	Present work	MZ470569	MZ470515
IIc	*Planaxis sulcatus*	CWR 106/09-2	Yemen, al-Hudeida	[19]	–	MT621368
IIc	*Planaxis sulcatus*	CWR 17/07-2	Yemen, Little Aden	[19]	–	MT621370
IIc	*Planaxis sulcatus*	MI26	Iran, Northern Persian Gulf	[28]	LC167817	–
IIc	*Planaxis sulcatus*	MI7	Iran, Persian Gulf	[28]	LC060527	–
IIc	*Planaxis sulcatus*	ZMB 107849-4	Oman, Sudh, Salahah	[19]	–	MT621375
IIc	*Planaxis sulcatus*	ZMB 117933-1	Egypt, Hurghada	[19]	MT587883	MT593025
IIc	*Planaxis sulcatus*	ZMB 117933-2	Egypt, Hurghada	[19]	MT620958	MT621384
III	*Planaxis sulcatus*	HVDBM_KZN_153	South Africa, Kosi Bay	BOLD	HVDBM722-12.COI-5P	–
III	*Planaxis sulcatus*	HVDBM_KZN_154	South Africa, Kosi Bay	BOLD	HVDBM723-12.COI-5P	–
III	*Planaxis sulcatus*	HVDBM_KZN_156	South Africa, Kosi Bay	BOLD	HVDBM725-12.COI-5P	–
III	*Planaxis sulcatus*	HVDBM_KZN_157	South Africa, Kosi Bay	BOLD	HVDBM726-12.COI-5P	–
III	*Planaxis sulcatus*	MNHN-IM-2009-24494	Mozambique, Xixuane, INHACA, MM11, 25° 59.5′ S, 32° 55.9′ E	Present work	MZ470570	MZ470516
III	*Planaxis sulcatus*	MNHN-IM-2009-24509	Mozambique, Xixuane, INHACA, MM11, 25° 59.5′ S, 32° 55.9′ E	Present work	MZ470571	MZ470517
III	*Planaxis sulcatus*	MNHN-IM-2009-24513	Mozambique, Xixuane, INHACA, MM11, 25° 59.5′ S, 32° 55.9′ E	Present work	MZ470572	–
III	*Planaxis sulcatus*	MNHN-IM-2009-24514	Mozambique, Ponta Torres, INHACA, MM3, 26° 03.9′ S, 32° 57.3′ E	Present work	MZ470573	MZ470518
III	*Planaxis sulcatus*	MNHN-IM-2009-24530	Mozambique, Ponta Torres, INHACA, MM3, 26° 03.9′ S, 32° 57.3′ E	Present work	MZ470574	–
III	*Planaxis sulcatus*	MNHN-IM-2009-27034	Madagascar, Ilot de Lokaro, ATIMO VATAE, TM5, 24° 56.5′ S, 47° 07.1′ E	Present work	MZ470575	MZ470519
III	*Planaxis sulcatus*	MNHN-IM-2009-27049	Madagascar, crique au NE phare d′ Evatra, ATIMO VATAE, TM8, 24° 58.7′ S, 47° 05.9′ E	Present work	MZ470576	MZ470520
III	*Planaxis sulcatus*	MNHN-IM-2009-27101	Madagascar, Ambatobe, Bavarama, ATIMO VATAE, BM06, 25° 27.9′ S, 44° 57.6′ E, 1 m	Present work	MZ470577	MZ470521
III	*Planaxis sulcatus*	MNHN-IM-2009-27120	Madagascar, Ambatobe, Bavarama, ATIMO VATAE, BM06, 25° 27.9′ S, 44° 57.6′ E, 1 m	Present work	MZ470578	MZ470522
III	*Planaxis sulcatus*	MNHN-IM-2009-27342	Mozambique, Ponta Torres, INHACA, MM3, 26° 03.9′ S, 32° 57.3′ E	Present work	MZ470579	MZ470523
III	*Planaxis sulcatus*	MNHN-IM-2009-27343	Mozambique, Ponta Torres, INHACA, MM3, 26° 03.9′ S, 32° 57.3′ E	Present work	MZ470580	MZ470524
III	*Planaxis sulcatus*	MNHN-IM-2009-27344	Mozambique, Xixuane, INHACA, MM11, 25° 59.5′ S, 32° 55.9′ E	Present work	MZ470581	MZ470525
III	*Planaxis sulcatus*	MNHN-IM-2009-27345	Mozambique, Xixuane, INHACA, MM11, 25° 59.5′ S, 32° 55.9′ E	Present work	MZ470582	MZ470526
III	*Planaxis sulcatus*	UF 423448	Madagascar, Nosy Ankazoberavina, S peninsula, near Nosy Be, 13° 29′ 21″ S , 47° 58′ 36″ E	Present work	MZ470583	MZ470527
III	*Planaxis sulcatus*	ZMB 106003-h1	Indonesia, Southeast Sulawesi, Muna Island, Raha, beach nr Napapale Lagoon, 04° 54.190′ S, 122° 45.430′ E	[19]	MT620951	–
III	*Planaxis sulcatus*	ZMB 106376-h2	Fiji, Musket Cove, Malololailai Island, 17° 46.260′ S, 177° 11.830′ E	[19]	MT620952	–
III	*Planaxis sulcatus*	ZMB 107933-1	Thailand, Gulf of Siam, E coast of Koh Phangan, Ao Thong Nai Pan Noi, 9° 46.883′ N, 100° 3.355′ E	[19]	MT620954	–
III	*Planaxis sulcatus*	ZMB 117931-1	Mauritius, Rodrigues, Baie Topaze	[19]	–	MT62138
III	*Planaxis sulcatus*	ZMB 117931-2	Mauritius, Rodrigues, Baie Topaze	[19]	MT587884	MT593026
III	*Planaxis sulcatus*	ZMB 117932-1	Mozambique, Mocimboa da Paria	[19]	-	MT621382
III	*Planaxis sulcatus*	ZMB 117932-2	Mozambique, Mocimboa da Paria	[19]	MT620957	MT621383
III	*Planaxis sulcatus*	ZMB 117936-1	Madagascar, Ste Marie Island	[19]	MT620959	MT621385
III	*Planaxis sulcatus*	ZMB 127569a-1	Thailand, North Khao Lak, Laem Pakarang, 8° 44.171′ N, 98° 13.535′ E,	[19]	MT620960	MT621387
III	*Planaxis sulcatus*	ZMB 127569b-1	Thailand, North Khao Lak, Laem Pakarang, 8° 44.171′ N, 98° 13.535′ E,	[19]	MT620961	MT621388
III	*Planaxis sulcatus*	ZMB 127569b-2	Thailand, North Khao Lak, Laem Pakarang, 8° 44.171′ N, 98° 13.535′ E,	[19]	MT620962	MT621389
–	*Planaxis* sp.	AF1	French Polynesia, Afareaitu, Moorea Island, 17° 19′ 49.2″ S, 149° 28′ 26.8″W,	[29]	KT149309	–
–	*Planaxis planicostatus*	ZMB 108261-h1	Panama, Paitilla, Bay of Panama, Pacific	[19]	–	MT621366
–	*Supplanaxis niger*	ZMB 117939-1	Madagascar, Southeast Ste Marie Island	[19]	MT587879	MT593021
–	*Supplanaxis niger*	ZMB 117946-1	Indonesia, Sumatra, Aceh, Ule-le	[19]	MT587878	MT593020

### Molecular analyses and sequence alignment

Thirty-nine of the 55 specimens sequenced for this study were obtained during expeditions organized by the MNHN and Pro-Natura International as part of the *Our Planet Reviewed* program. These specimens were anesthetized using magnesium chloride (MgCl_2_) or were microwaved to separate the animal from the shell [30]. Tissue clips of foot tissue were preserved in 95–98% ethanol. Specimens in the USNM were cracked and preserved whole in 95% ethanol.

Two labs (USNM; Sapienza University of Rome) contributed sequences for this study using slightly different protocols. At the USNM, whole genomic DNA was extracted from a ~ 1 mm^3^ tissue clip of the foot using an Autogenprep965 (Autogen, Holliston, MA) automated phenol:chloroform extraction with a final elution volume of 50 µL. A 691 base pair (bp) fragment of cytochrome c oxidase subunit I (COI) was amplified using the jgLCOI primer [31] in combination with Cerithioid_COIR [32]; a 505–508 bp fragment of 16S ribosomal DNA was amplified with the universal 16S AR/BR primers [33]. PCR reactions were performed with 1 µL of undiluted DNA template in 20 µL reactions. Reaction volumes for COI consisted of 10 µL of Promega Go-Taq Hotstart Master Mix, 0.15 µM each primer, 0.25 µg/µL BSA, 1.25% DMSO and an amplification regime of an initial denaturation at 95 °C for 7 min, followed by 45 cycles of denaturation at 95 °C for 45 s, annealing at 42 °C for 45 s, extension at 72 °C for 1 min and a final extension at 72 °C for 3 min. Reaction volumes for 16S were 1x Biolase (Bioline, Taunton, MA) reaction buffer, 500 µM dNTPs, 3 mM MgCl_2_, 0.15 µM each primer, 0.25 µg/µL BSA, 1 unit Biolase DNA polymerase and an amplification regime of initial denaturation at 95 °C for 5 min, followed by 35 cycles of denaturation at 95 °C for 30 s, annealing at 48 °C for 30 s and extension at 72 °C for 45 s, followed by a final extension at 72 °C for 5 min. PCR products were purified using the ExoSAP-IT protocol (USB Corporation). BigDye 3.1 (ABI, Foster City, CA) sequencing reactions and sequencing on an ABI 3730XL DNA analyzer capillary array were done following manufacturer’s instructions. At Sapienza University of Rome, whole genomic DNA was extracted from a ~ 1 mm^3^ tissue clip of foot tissue by using a ‘salting out’ protocol [34], with a final elution of 50 µL. A 658 bp of COI was amplified using the jgLCOI and jgHCO primers [31]; a ~ 800 bp fragment of 16S ribosomal DNA was amplified with the 16SA [35] and CGLeuR [36] primers. PCR reactions were performed with 1 µL of undiluted DNA template in 25 µL reactions. Reaction volumes consisted of 2.5 µL of 10x NH4 Reaction Buffer, 2.5 µL of 50 mM MgCl_2_ Solution, 0.15 µL of BIOTAQ DNA Polymerase, 0.4 µL of each 25 pM primer solution, 1 µL of 10% BSA solution, 0.5 µL of 10 mM nucleotide mix solution. PCR conditions for COI followed a “touchdown” profile as in [37], while for 16S were as follows: initial denaturation (94 °C/4′); 35 cycles of denaturation (94 °C/30''), annealing (52 °C/40″), and extension (94 °C/1′); final extension (72 °C/10′). PCR products were purified using ExoSAP-IT (USB Corporation) and sequenced at Macrogen, Inc.

Amplicons were sequenced in both directions to ensure accuracy. Chromatograms were visually inspected and corrected as necessary in Geneious v. 11 (Biomatters). COI alignments were translated into amino acids to check for stop codons and frameshift mutations, then trimmed to 658 bp. 16S rRNA sequences were aligned with MAFFT v. 7 [38, 39] using the E-INS-i algorithm which performed better on our expanded dataset than the Q-INS-i algorithm used by Wiggering et al. [19] in aligning variable regions. All newly generated sequences have been deposited in GenBank. See Table 1 for sequence accession numbers and voucher information.

### Species delimitation analyses

The final dataset for *Planaxis sulcatus* comprised 88 COI and 81 16S rRNA sequences. Primary Species Hypotheses (PSHs) (*sensu* [40, 41]) were formulated using ASAP [42] for the COI dataset (default parameters) with a distance matrix constructed in PAUP* v.4.0 [43] using the best-fit nucleotide substitution model (GTR + I + G) calculated with jModelTest v. 2 [44]. ASAP is the updated implementation of the ABGD hierarchical clustering algorithm [42] which has the added functionality of calculating a so-called “ASAP-score” that is used to rank the alternative partitioning schemes. It also has the benefit of obviating the need in ABGD for an a priori, user-defined range of *P* values which correspond to the minimum and maximum intraspecific divergence. Wiggering et al. defined P_min_ = 0.0031 and P_max_ = 0.041 based on COI distance values observed in a distantly related gastropod genus, *Littoraria* (Littorinoidea).

PSHs were then tested for reciprocal monophyly [45, 46] through phylogenetic analysis on the combined dataset (COI partitioned by codon position + 16S rRNA), using Bayesian (BA) and maximum likelihood (ML) methods. Because our dataset includes a substantial number of additional specimens compared with that of Wiggering et al., we re-estimated the best-fit nucleotide substitution models. Nucleotide substitution models were selected using jModelTest and were as follows: COI 1^st^ codon position = SYM + I; COI 2nd codon position = F81; COI 3^rd^ codon position = GTR + G; 16S rRNA = GTR + I + G. Bayesian analyses were run using MrBayes v. 3.2 [47] on the CIPRES Science Gateway v. 3.3 [48] (10^7^ generation, 25% burn-in). MCMC convergence was assumed to be reached when the effective sample size was >200 and values of the potential scale reduction factor were 1 (analysed with Tracer v. 1.7 [49]). ML analyses were run online using W-IQ-TREE v. 1 [50] (ultrafast bootstrap replicates = 1000). A node was considered supported if the corresponding Bayesian posterior probability (PP) was ≥0.95. The same threshold was used for interpreting the ultrafast bootstrap value (UFb) obtained from the ML analysis, as suggested by the authors of IQ-TREE [51].

PSHs were further explored with bGMYC [52], a Bayesian implementation of the general mixed Yule-coalescent model for species delimitation, performed on the COI dataset using the bGMYC package in R v. 3.2.1 [53]. Ultrametric trees were generated using BEAST v.1.8 [54] (2 runs of 20^8^ generations, sampled each 1000, 25% burn-in, substitution model = HKY, clock model = lognormal relaxed clock, tree prior = Birth-Death Process). MCMC convergence was assessed with Tracer 1.7 [49] and assumed to have occurred if effective sample sizes were greater than 200. The bGMYC analysis was run on 100 trees sampled equidistantly from those obtained from the BEAST analysis (generations = 50000, burn-in=40000, t1 = 1, t2 = 88, thinning = 100).

COI pairwise genetic distances were calculated using MEGA v.7 [55] (nucleotide substitution model = Kimura 2-parameter, pairwise deletion of missing data). Median-joining [56] (MJ) haplotype networks were inferred and visualized using PopART (http://popart.otago.ac.nz).

## Results

### Species delimitation

**Fig. 2.**
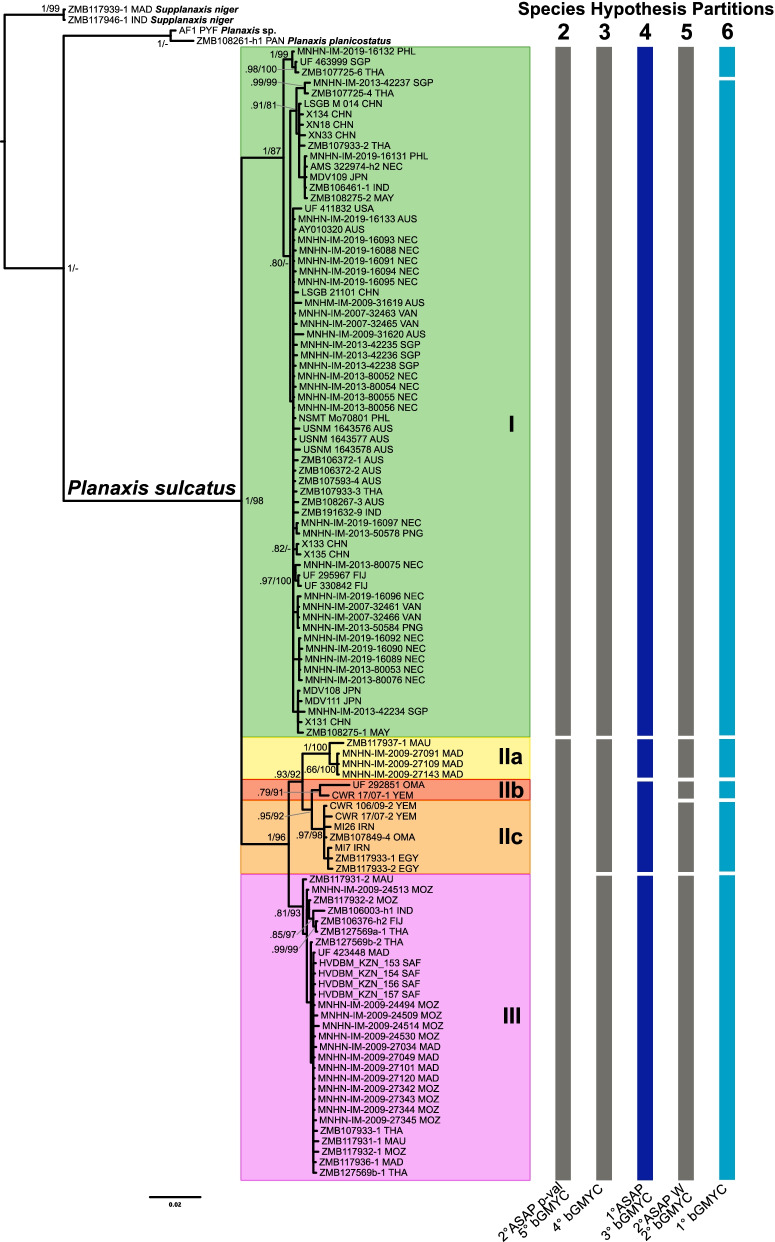
Bayesian phylogram based on the COI+16S rRNA dataset. Numbers at the nodes are PP and UFb support values, respectively (only values higher than 0.65 are reported). Roman numbers indicate clade names. Bars on the right indicate the primary species hypothesis partitions identified by ASAP and bGMYC analyses (dark blue = best ASAP partition; light blue = best bGMYC partition). Numbers at the top of the bars indicate the number of species for each partition; at the bottom of the bars are the partition rankings for both programs (p-val = ASAP P-rank; W = ASAP relative gap width score). Abbreviations of specimen collection countries: *AUS* Australia, *CHN* China, *EGY* Egypt, *FIJ* Fiji, *IND* Indonesia, *IRN* Iran, *JPN* Japan, *MAD* Madagascar, *MAU* Mauritius, *MAY* Malaysia, *MOZ* Mozambique, *NEC* New Caledonia, *OMA* Oman, *PAN* Panama, *PHL* Philippines, *PNG* Papua New Guinea, *PYF* French Polynesia, *SAF* South Africa, *SGP* Singapore, *THA* Thailand, *USA* United States of America, *VAN* Vanuatu, *YEM* Yemen

**Fig. 3.**
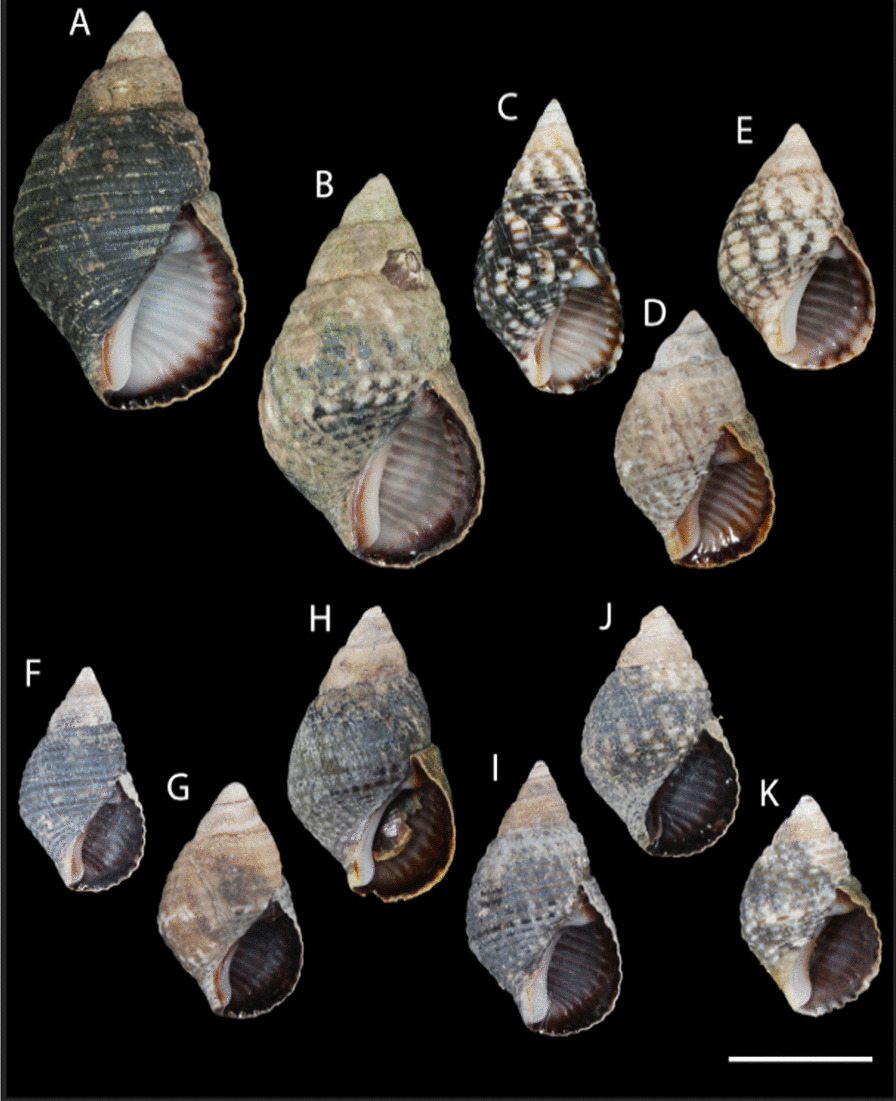
Shell variation in *Planaxis sulcatus*. Clade I, A-E; Clade IIa, F-G; Clade IIb, H; Clade III, I-K; **A** Changi Coast, Singapore, MNHN-IM-2013-42235. **B** North New Caledonia, MNHN-IM-2019-16090. **C** Espiritu Santo, Vanuatu. MNHN-IM-2007-32461. **D** Viti Levu, Fiji, UF 330842 (paravoucher). **E** Port Hedland, Australia, MNHN-IM-2009-31620. **F** South Madagascar, MNHN-IM-2009-27143. **G** South Madagascar, MNHN-IM-2009-27091. **H** Masirah Island, Oman, UF 292851. **I** South Madagascar, MNHN-IM-2009-27120. **J** Inhaca Island, Mozambique, MNHN-IM-2009-24509. **K** Near Nosy Be, Madagascar, UF 423448. All specimens represent sequenced vouchers except 3D. Scale, 1 cm

The 10 best partitions found by ASAP divided the COI dataset into 2 to 56 hypothetical species; no partition included all specimens in a single species. The best ASAP partition divided the dataset into four PSHs, corresponding to Clades I, IIa, IIb+IIc, and III (Figs. 2, 3, and Additional file 1: Fig. S1; Table 1; clade names correspond to those used by Wiggering et al.). This was the best partition according to both ASAP P-rank and W score (5.22e^−03^ and 2.30e^−03^, respectively). The second-best partition according to the P-rank score divided the dataset into two PSHs (Clades I and II+III), while the second-best according to the W score identified five PSHs (Clades I, IIa, IIb, IIc, and III). All subsequent partitions identified between 25 and 56 PSHs. The bGMYC analysis (Fig. 2 and Additional file 1: Fig. S2) recovered five partitions that divided the dataset into two to six groups, with increasing posterior probability values. The partition with the highest PP value (PP=0.4) divided the COI dataset into six groups, considering specimens MNHN-IM-2019-16132, UF 4639999, and ZMB107725-6 as an entity distinct from Clade I. The other partitions divided the dataset into the same clades as found by ASAP

All phylogenetic analyses supported monophyly of *Planaxis sulcatus* sensu lato (Fig. 2, Additional file 1: Figs. S3–S6; PP=1 UFb=98). In analyses of the concatenated COI+16S rRNA dataset, monophyly of Clades II+III (PP=1 UFb=96), IIa (PP=1 Ufb=100), and IIc (PP=0.97 UFb=98) was supported by both BA and ML analyses. Monophyly of Clades I and IIb+IIc was supported by only one of the two methods (PP=1 and PP=0.95, respectively), while that of Clade IIb and III was not supported (PP=0.79 UFb=91, and PP=0.81 UFb=93, respectively). The BA tree resulting from analysis of the COI dataset recovered two reciprocally monophyletic clades (I and II+III, both PP=1), while all other single-gene analyses with BA or ML did not support any of the other subdivisions within *P. sulcatus*.

COI genetic distances within and between clades were calculated using K2P model-corrected distances (Table 2). For the two-PSH partition, the range of intra-clade genetic distances was 0**–**6.19%, and 6.32**–**12.09% between clades. For the four- and five-PSH partitions, the ranges of intra-clade genetic distances were 0**–**4.02% and 0–2.56% respectively, while the ranges of inter-clade distances were almost identical for both (3.69**–**12.09% and 3.68**–**12.09%, respectively). The range of genetic distances when all *P. sulcatus* specimens were considered to belong to a single species was 0**–**12.09%.

**Table 2:** *Planaxis sulcatus* intra- and inter-clade K2P model-corrected genetic distances (percent) in COI

Clade	I	IIa	IIb	IIc	IIb+IIc	III	II+III
I	**0–2.56**						
IIa	7.9**–**10.39	**0**					
IIb	8.69**–**10.19	5.56	**na**				
IIc	8.73**–**12.09	4.03**–**4.68	3.68**–**4.02	**0.36–1.18**			
IIb+IIc	8.69**–**12.09	4.03**–**5.56	na	na	**0.36–4.02**		
III	6.32**–**9.87	4.11**–**5.57	3.94**–**5.56	3.69**–**6.19	3.69**–**6.19	**0–1.73**	
II+III	6.32**–**12.09	na	na	na	na	na	**0–6.19**

*na* value not calculable; intra-clade ranges highlighted in bold

**Fig. 4:**
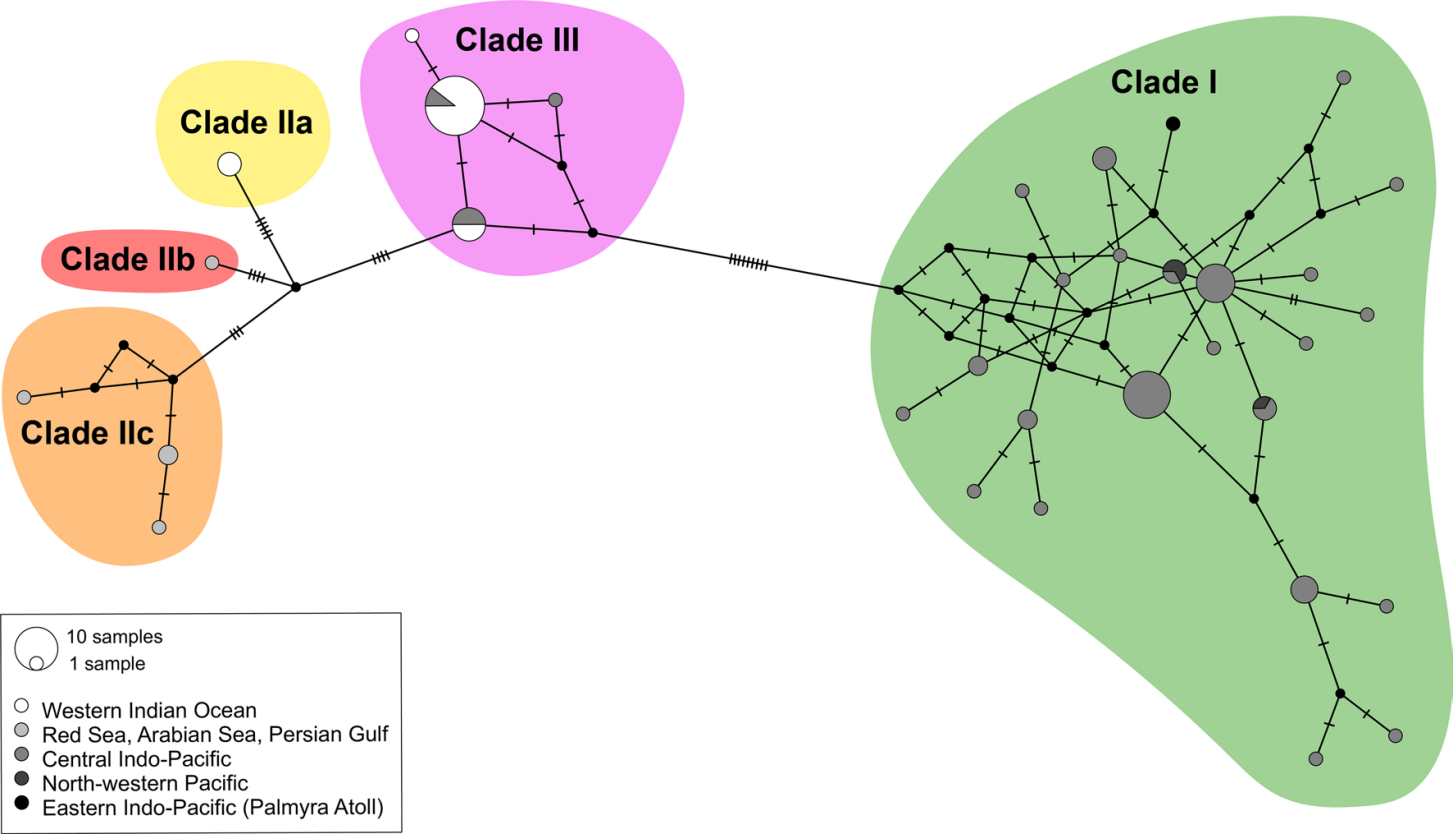
Median-joining COI haplotype network of *Planaxis sulcatus* specimens. Roman numbers and colours correspond to phylogenetic clades (as in Figs. 1 and 2; see text)

The MJ network analysis divided *P. sulcatus* haplotypes into two macro haplogroups corresponding to Clades I and II+III (Fig. 4), separated by at least 13 polymorphic sites. Haplotypes corresponding to Clades IIa, IIb, IIc, and III were each separated by between 8 to 9 polymorphic sites.

## Discussion

### One, two, or five species?

Our goal is not to resolve with certainty the number of distinct evolutionary lineages that comprise the *Planaxis sulcatus* complex. This will require an integrative taxonomic approach including detailed morphological (shell, radula) comparisons of name-bearing types in historical collections with the sequenced specimens. However, it seems certain that the evidence supports the interpretation that *P. sulcatus* comprises at least two evolutionary lineages, and quite probably more than that. The existence of at least two distinct lineages comprising Clade I (Central Indo-Pacific, North-western Pacific, and Palmyra Atoll), and Clade II (Red Sea, Arabian Sea, Persian Gulf, and Western Indian Ocean) + Clade III (Western Indian Ocean, and Central Indo-Pacific) was supported by the second-best ASAP P-rank score, one of the bGMYC partitions, reciprocal monophyly in phylogenetic analyses of the COI and COI+16S rRNA datasets, and, the MJ network of COI sequences that identified two major distinct haplogroups.

In the Wiggering et al. [19] analysis, ABGD and bGMYC converged on the interpretation that three evolutionary lineages are present in their dataset, corresponding to Clades I**,** II and III. Here we have added sequences for 77 individuals, more than tripling the size of the original dataset, and have expanded the geographic coverage to include southern Madagascar, South Africa, Western Australia, Singapore, China, Japan, Papua New Guinea, Philippines, Vanuatu and Palmyra. Interestingly, this tripartite subdivision is neither considered as the best partition by any of the species delimitation analyses of the expanded molecular dataset, nor supported by the phylogenetic analyses (the monophyly of Clade III was not supported).

Wiggering et al. [19] reported a maximum K2P model-corrected divergence in COI of 3.08% and interpreted it as indicative of intraspecific population structuring. However, this value is the result of a misinterpretation of the ABGD methodology. ABGD uses a range of prior intraspecific divergences (*P*; also referred to as prior maximal distances) to partition a dataset into putative species based on a statistically inferred barcode gap. ABGD uses a value of *P* within a user-defined range, which Wiggering et al. specified as *P*_min_ = 0.0031 and *P*_max_ = 0.0411, and searches for a barcoding gap above this threshold. Thus, the value of 3.08% reported in Wiggering et al. (Table S4) is the prior maximal distance that yields a single-species partition, and not the maximum K2P intraspecific divergence that can be calculated for the dataset. In fact, we recalculated a maximum value of 11.6% for the Wiggering et al. dataset, which is more consistent with the value of 12.09% obtained here for the expanded dataset. Both of these upper values far exceed what is considered as indicative of maximum species-level divergence for other marine caenogastropods (~ 2.2%-4.6%) [27, 57–59]. Even when considering the two-PSH partition recognizing Clades I and II+III, the maximum intra-clade genetic divergence (6.19%) still exceeds this threshold. The bGMYC analysis suggested the presence of up to six hypothetical species; however, none of the other methods used for the species delimitation analysis supported this hypothesis, and it may simply reflect the known tendency of bGMYC to oversplit the molecular dataset [60–62]. Our analyses produced two additional hypotheses, even if less supported, with four or five PSHs, identified as Clades I, IIa, IIb+IIc, and III (Fig. 2). These hypotheses were the best- and second best-scoring partitions according to ASAP P-rank and W score and yielded maximum intra-clade genetic distances in COI of 4.02% and 2.56%, respectively, still relatively high, but more consistent with hypothesized species-level thresholds for marine caenogastropods. The MJ haplotype network produced haplogroups that are less differentiated but still distinct. Moreover, high levels of inter-clade sequence divergence are maintained even where members of different clades occur in sympatry. For example, MNHN-IM-2009-27091 (Clade IIa), MNHN-IM-2009-27101 and MNHN-IM-2009-27120 (Clade III), collected at the same site within 100 m of each other in south Madagascar, differ by 5.02% sequence divergence in COI; ZMB107725-4 (Clade I) and ZMB107933-1 (Clade III), also collected at the same site, differed by 8.93%. In Indonesia, ZMB106461-1 and ZMB106003-h1, also from Clades I and III respectively, while not syntopic, were collected from two sites only ~10 km apart on Muna Island and differed by 7.49%. Sympatry of specimens from genetically divergent clades, for which there is no concordance in the results of different delimitation methods, as in the present case, is generally considered a diagnostic criterion for species delimitation (e.g. [63]). Even if some of our PSHs were not uniformly corroborated by the phylogenetic analyses, with some of the clades statistically supported only by one of the two methods in analyses of the concatenated dataset and were not always reciprocally monophyletic in the single-gene analyses, still the levels of genetic differentiation in sympatry suggest the presence of more than two species. The lack of support for Clades II and IIb, in particular, is undoubtedly a consequence of the comparatively poor sampling and the quantity of missing data; only 13 individuals were sequenced in Clade II and its subclades, and only four individuals were sequenced for both markers.

### The case for poecilogony

Wiggering et al. stated that, even if *Planaxis sulcatus* comprised a complex of cryptic species, their claim of poecilogony is upheld in Clade III, for which they documented individuals bearing both veligers and juveniles. However, even here the evidence seems inconclusive.

Clade III is distributed in the Indian Ocean and western Pacific and, in the analysis of Wiggering et al., included 12 sequenced specimens from Indonesia, Fiji, Thailand, Mozambique, Madagascar, and Mauritius. However, inferences of developmental mode were based on observations of 12 gravid individuals, none of which were sequenced, and only some of which can be considered unambiguous.

The fact that none were sequenced is problematic given that five of the observations were from Indonesia, an area of overlap between Clades I and III. With no apparent way to differentiate morphologically between adults of different clades, it is not clear how mode of development was assigned to the sequenced terminals. Indeed, all observations of developmental mode must be considered suspect in the region of overlap between the two clades (i.e., Indonesia, Thailand, Fiji). In the case of Indonesia, this issue is of little consequence given that all examined gravid females had at most late larvae in their brood pouch and all terminals were inferred to possess veligers. In the case of Fiji, the terminal was coded based on the dissection of one gravid female, however, the expanded molecular analysis indicates that clades I and III may overlap in this area as well. Thus, it is not clear to which clade this developmental mode should be assigned.

Some geographic areas were not represented by gravid females, chief among them being Thailand and Madagascar. In these cases, Wiggering et al. “assumed reproductive mode based on area of origin.” This led to inferences of developmental mode for individuals in their tree that were not empirically based, and in some cases conflicted with observations. For example, this coding strategy resulted in two individuals collected at the same site in Thailand (Clade I, ZMB107725-4; Clade III, ZMB107933-1) being assigned to different developmental modes, veligers and juveniles, respectively, despite the fact that no gravid females were examined from Thailand. For Mauritius, three terminals from Clade III were coded with two different developmental modes (ZMB117931-2, ZMB117937 as veligers, and ZMB117931-1, as juveniles), with observations of only a single gravid female bearing eggs/early larvae. For Mozambique, four gravid females were observed, three with larvae and one with juveniles, none of which were sequenced, but both sequenced terminals were inferred to possess juveniles. This inference was apparently strengthened by one observation of a gravid female from Tanzania ostensibly bearing juveniles <0.5 mm, also with no corresponding sequence. However, the specimen examined from Tanzania (ZMB 108265-15), listed in Wiggering et al. Table S3 as bearing large numbers (6980) of juveniles, is figured in their Fig. 1c as a late larva.

Observations of larvae in Clade III included one individual from Fiji, five from Indonesia, one from Mauritius, and three from Mozambique. The six observations from Fiji and Indonesia were obtained from unsequenced individuals collected in the area of overlap between Clades I and III, and potentially, in the case of Fiji, from a mislocalized specimen (see “Future research”, below). Thus, these observations should be considered unreliable. This leaves four observations from Mauritius and Mozambique. The adage, *absence of evidence is not evidence of absence*, pertains here. As acknowledged by Wiggering et al., an observation of larvae in the brood pouch is inconclusive with regard to mode of development given the possibility that the individual was sampled early in the brooding phase. For the two observations of juveniles, one of them is erroneous, leaving the entire case of poecilogony in Clade III to rest on a single observation from Mozambique which was extrapolated to five terminals on their tree, sometimes in direct contradiction to observations of gravid females from the same geographic area.

Thus, the coding strategy used by Wiggering et al. [19] to assign developmental mode to terminals in their tree extrapolated inconsistently from a few observations for mostly unsequenced individuals; this made their tree difficult to interpret at best, and misleading at worst. Their claim that “developmental modes do not entirely correlate with genetic clades” (Wiggering et al. [19]: 5) does not carry much weight.

### Future research

As outlined by Knott & McHugh [20], the first step in identifying reliable cases of poecilogony is to convincingly rule out potentially cryptic species. Resolving this issue with certainty necessitates advancing several lines of investigation. The first will require determining the number of distinct evolutionary lineages and their rank within an integrative taxonomic framework. It is possible that morphological characters may come to light that allow discrimination of the molecular clades. For example, two recently identified molecular lineages formerly recognized as *Supplanaxis nucleus* in the Caribbean were shown through careful scrutiny to be morphologically diagnosable in features of the shell and radula [64]. Indeed, Bandel [65] observed differences in radular morphology of *Planaxis sulcatus* individuals from the Red Sea compared to those from South Africa [66], and others from New Caledonia [17], representing three different molecular clades in our tree. Bandel [65] noted that some of the differences were of so great a magnitude that they could be indicative of species-level differences.

A related line of enquiry will require refining geographical distributions and observations of developmental mode in the north-western Indian Ocean. Assuming that Clade III represents a distinct evolutionary lineage, as described above, the case for poecilogony made by Wiggering et al. in this clade rests on essentially just one observation of an individual from northern Mozambique bearing juveniles 0.6–1.0 mm in size (AMS 322969-8; Wiggering et al. Table S3) in a clade where all other observations were only of larvae. One potential explanation is that this represents an extension of Clade II outside the Red Sea and Persian Gulf. Barkati and Ahmed [67] reported ‘direct’ development in individuals from Karachi, Pakistan which may represent an extension of Clade II to the northern Arabian Sea. Another potential explanation is that all observations of larvae in the Indian Ocean were made on individuals early in the brooding phase. However, the situation in the north-western Indian Ocean is far more complex than the analysis of Wiggering et al. would suggest. Although the Red Sea population has been recognized as a distinct species by Dekker & Orlin [68] and Janssen et al. [69], it is not known how far the Red Sea form is distributed in the northern Indian Ocean and, further, not all Red Sea *Planaxis sulcatus* produce juveniles. As reported by Hulings [70] and Bandel [71], individuals from the Gulf of Aqaba release veligers. The presence of two developmental modes in the Red Sea may support the interpretation that two evolutionary lineages are represented here, as indicated by the five-PSH partition. Thus, the case for or against poecilogony in *Planaxis sulcatus* will depend on the resolution of clade structure and distribution of developmental mode in the north-western Indian Ocean.

It should be noted that we consider the record from Fiji reported in Wiggering et al. from Clade III (ZMB 106376 h2) to be problematic. Our analyses indicated that Clade I (UF 295967, UF 330842) occurs in Fiji, which is more consistent with the interpretation of a single widespread clade in the western Pacific. The presence of Clade III there would require a gap spanning the comparatively well sampled areas of South East Asia, northern Australia, New Caledonia, and Vanuatu. Although our geographical coverage is far from comprehensive, this seems unlikely even with present sampling effort. Thus, we consider their record from Fiji as dubious pending further study, possibly representing a mislocalized specimen or sequencing contamination.

Lastly, Wiggering et al. claimed there are no morphological differences among larvae in the brood pouch, regardless of inferred development type. It may be difficult to assess such differences through light microscopic analysis, but it should be possible to recognize differences in mode of larval nourishment from the size of the embryonic shells. This likely requires higher magnification and higher resolution (e.g., scanning electron microscopy) to assess. Moreover, while differences in the embryonic shells may be subtle, mature veliger larvae that have been brooded (Wiggering et al. [19]: Fig. 1d, E) are readily differentiable from free-swimming forms in size, ornament and number of whorls; differences in size and number of whorls are also evident among free-swimming veligers from different clades (see Houbrick [72]: fig. 1e; Bandel [71]: pl. 2, figs: 5, 7, 9). Unfortunately, adult planaxids do not retain undamaged protoconchs into adulthood given the unforgiving habitats where they live, and postlarval juveniles are rare in collections. Thus, documenting protoconch morphology from specimens in historical collections is a challenge. Future collecting strategies should include subadults and juveniles as possible.

## Conclusions

We conclude that the evidence for the existence of a single, widespread, species of *Planaxis sulcatus*, and for the presence of poecilogony in Clade III, is equivocal. The hypothesis that *Planaxis sulcatus* comprises at least two distinct evolutionary lineages was instead robust to the addition of a significant number of new sequences and to expanding the geographic coverage. Additional sampling of specimens is needed, especially in Clade II, as well as a broader comparative analysis of the protoconchs to assess morphological differences within and among the clades, particularly of the mature veliger stages, as part of an integrative taxonomic approach. Taxonomy of this complex would certainly benefit from the use of additional molecular markers, including nuclear ones (e.g., ribosomal intergenic spacers, microsatellites, RAD markers) to reach a more robust species delimitation.

## Supplementary Information

**Additional file 1.** Additional figures S1–S6.

## Data Availability

The genetic sequences generated during the current study are available in the GenBank repository (MZ470478–MZ470583). Permanent link to sequences: https://www.ncbi.nlm.nih.gov/nuccore/?term=MZ470528:MZ470583%5Baccn%5D

## Abbreviations

BOLD: Barcode of Life Data System
MNHN: Muséum national d’Histoire naturelle
USNM: National Museum of Natural History
UF: Florida Museum of Natural History
ZMB: Museum für Naturkunde
