# Supplementary material for: Re-evaluating the case for poecilogony in the gastropod Planaxis sulcatus (Cerithioidea, Planaxidae)
Source: BMC Ecol Evol. 2022 Feb 7;22:13. doi: 10.1186/s12862-022-01961-7 (PMC8822645; doi:10.1186/s12862-022-01961-7)
Supplement: Supplementary file 1 — Additional file 1. Additional figures S1–S6. [file 12862_2022_1961_MOESM1_ESM.pdf]

Re-evaluating the case for poecilogony in the gastropod *Planaxis sulcatus* (Cerithioidea, Planaxidae)

Giulia Fassio[1,2], Philippe Bouchet[3], Marco Oliverio[2], and Ellen E. Strong[4*]

[1]Department of Biology and Biotechnologies "Charles Darwin", Sapienza University of Rome, Zoology – Viale dell'Università 32, 00185 Rome, Italy
[2]Department of Biology and Evolution of Marine Organisms, Stazione Zoologica Anton Dohrn, Villa Comunale, 80121 Naples, Italy
[3]Institut de Systématique, Évolution, Biodiversité ISYEB–UMR 7205–CNRS, MNHN, UPMC, EPHE, Muséum National d'Histoire Naturelle, Sorbonne Université, Paris, France
[4]Department of Invertebrate Zoology, National Museum of Natural History, Smithsonian Institution, Washington, DC, 20013, USA
*corresponding author: STRONGE@si.edu

Supplementary Materials

Table of Contents:

ASAP analysis on the COI dataset	**Figure S1**
Pairwise posterior probability matrix calculated by bGMYC on the COI dataset	**Figure S2**
Bayesian phylogenetic inference on the COI dataset	**Figure S3**
Maximum Likelihood phylogenetic inference on the COI dataset	**Figure S4**
Bayesian phylogenetic inference on the 16S dataset	**Figure S5**
Maximum Likelihood phylogenetic inference on the 16S dataset	**Figure S6**

Nb of species	asap-score	P-val (rank)	W (rank)	Treshold dist.	Text
* 4	1.00	5.22e-03 (1)	2.30e-03 (1)	0.044058	list csv
2	3.00	2.13e-02 (2)	7.04e-04 (4)	0.068785	list csv
* 5	3.00	3.83e-01 (4)	1.93e-03 (2)	0.027700	list csv
* 4	7.50	7.68e-01 (12)	8.08e-04 (3)	0.046489	list csv
25	12.50	3.13e-01 (3)	2.27e-05 (22)	0.003419	list csv
49	13.00	4.61e-01 (6)	4.29e-05 (20)	0.001552	list csv
56	13.50	6.01e-01 (8)	4.29e-05 (19)	0.001546	list csv
51	16.00	8.12e-01 (14)	4.29e-05 (18)	0.001552	list csv
52	16.00	8.16e-01 (15)	4.29e-05 (17)	0.001552	list csv
53	16.00	8.18e-01 (16)	4.29e-05 (16)	0.001549	list csv

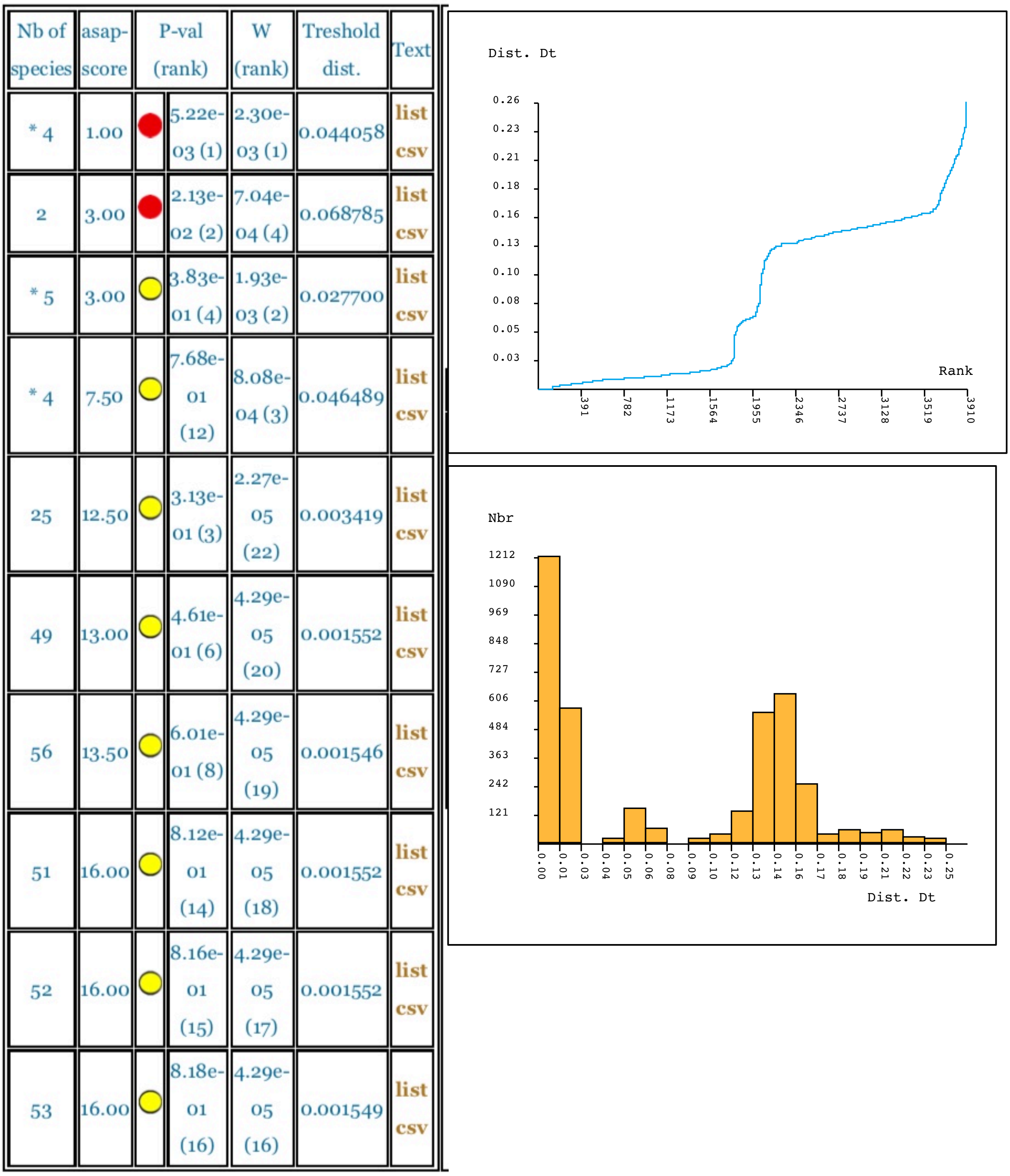

Figure S1. ASAP analysis of the COI dataset. Left, ASAP ten best partitions; upper right, ASAP ranked genetic distances; lower right, ASAP histogram of genetic distances.

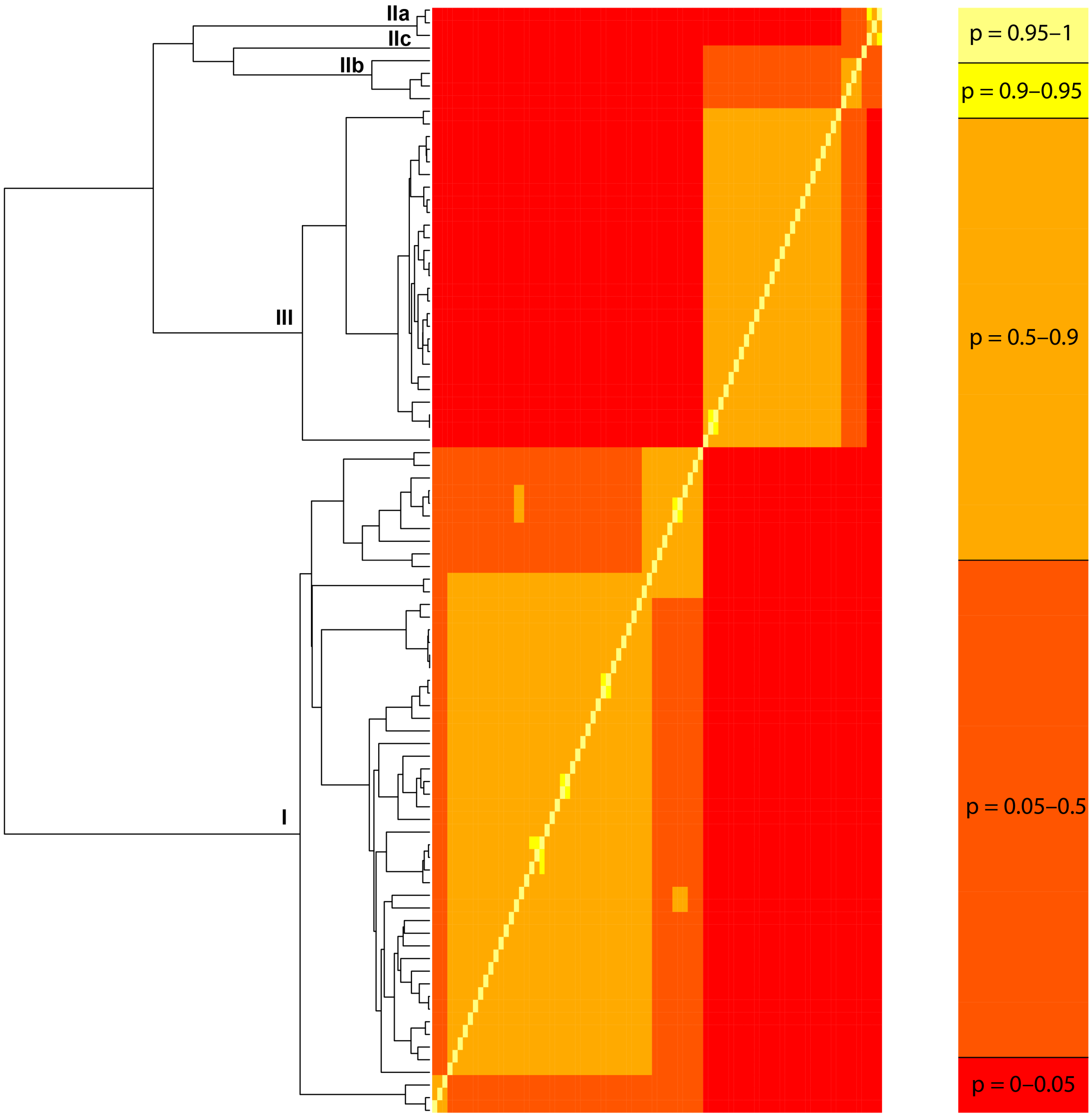

Figure S2. Pairwise posterior probability matrix calculated by bGMYC on the COI dataset, visualized as a heat map on the BEAST ultrametric tree. Colours correspond to the value of the posterior probabilities of conspecificity calculated for each specimen pair according to the scale on the right. Block heights are proportional to the width of the range that they represent.

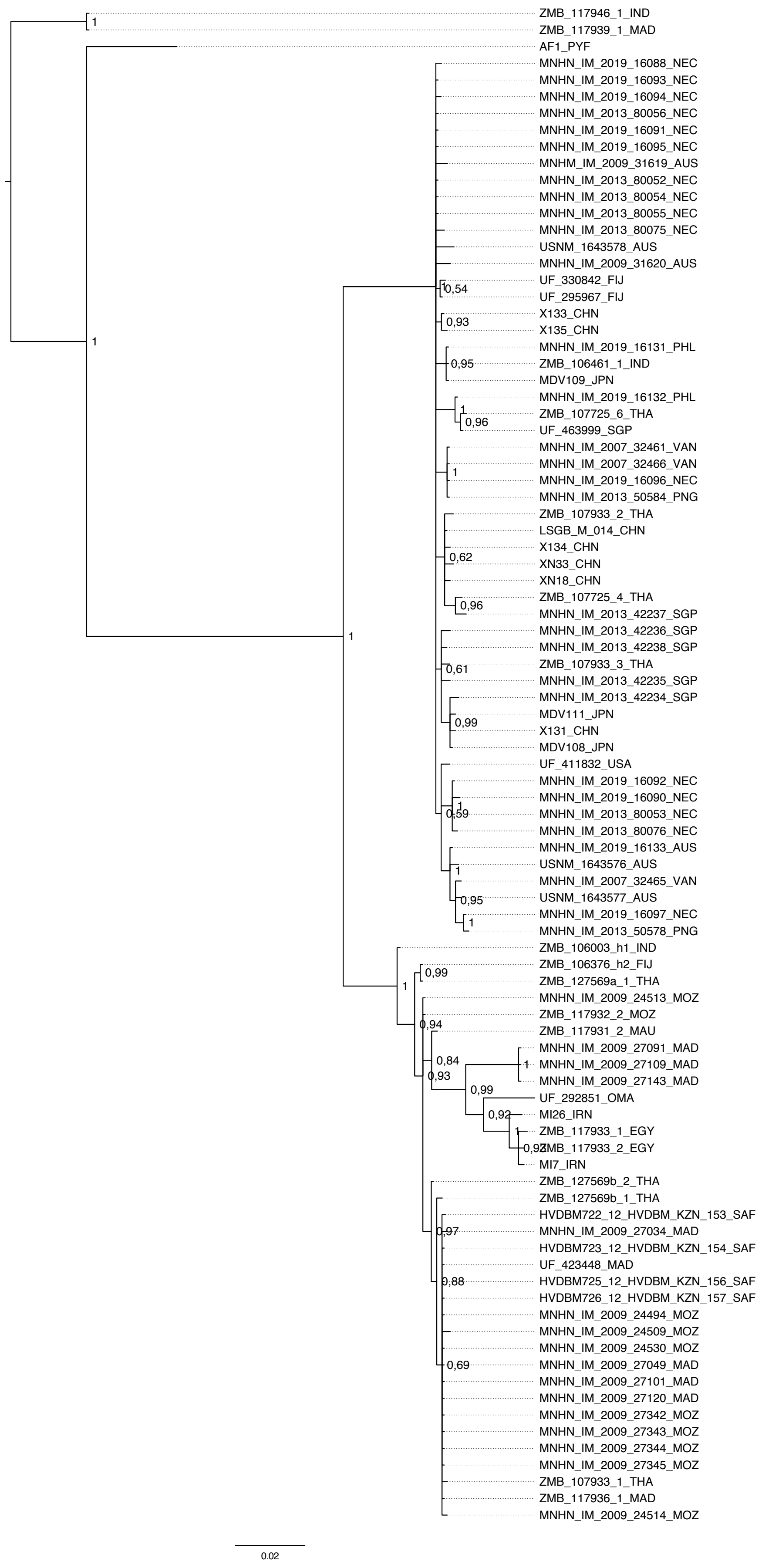

Figure S3. Bayesian tree based on the COI dataset.

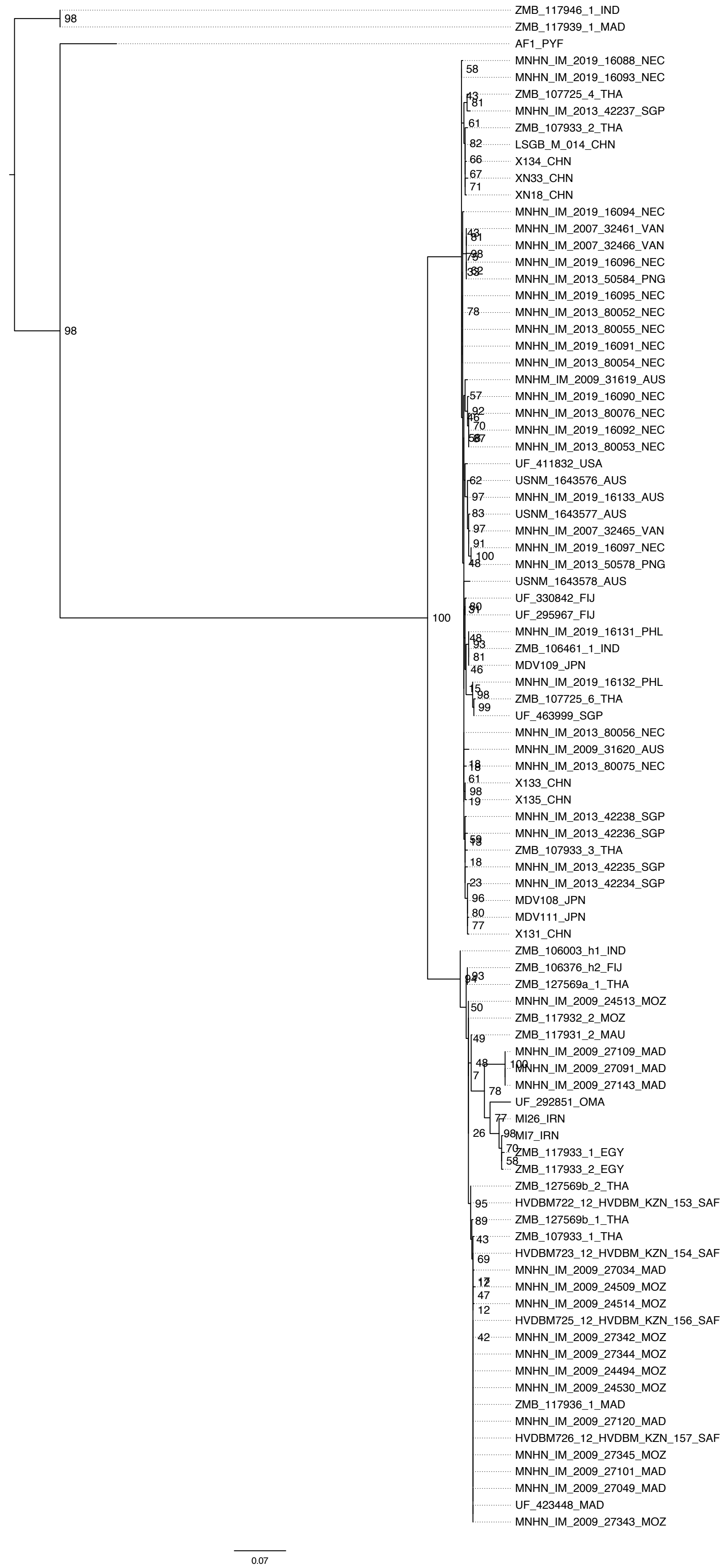

Figure S4. Maximum Likelihood tree based on the COI dataset.

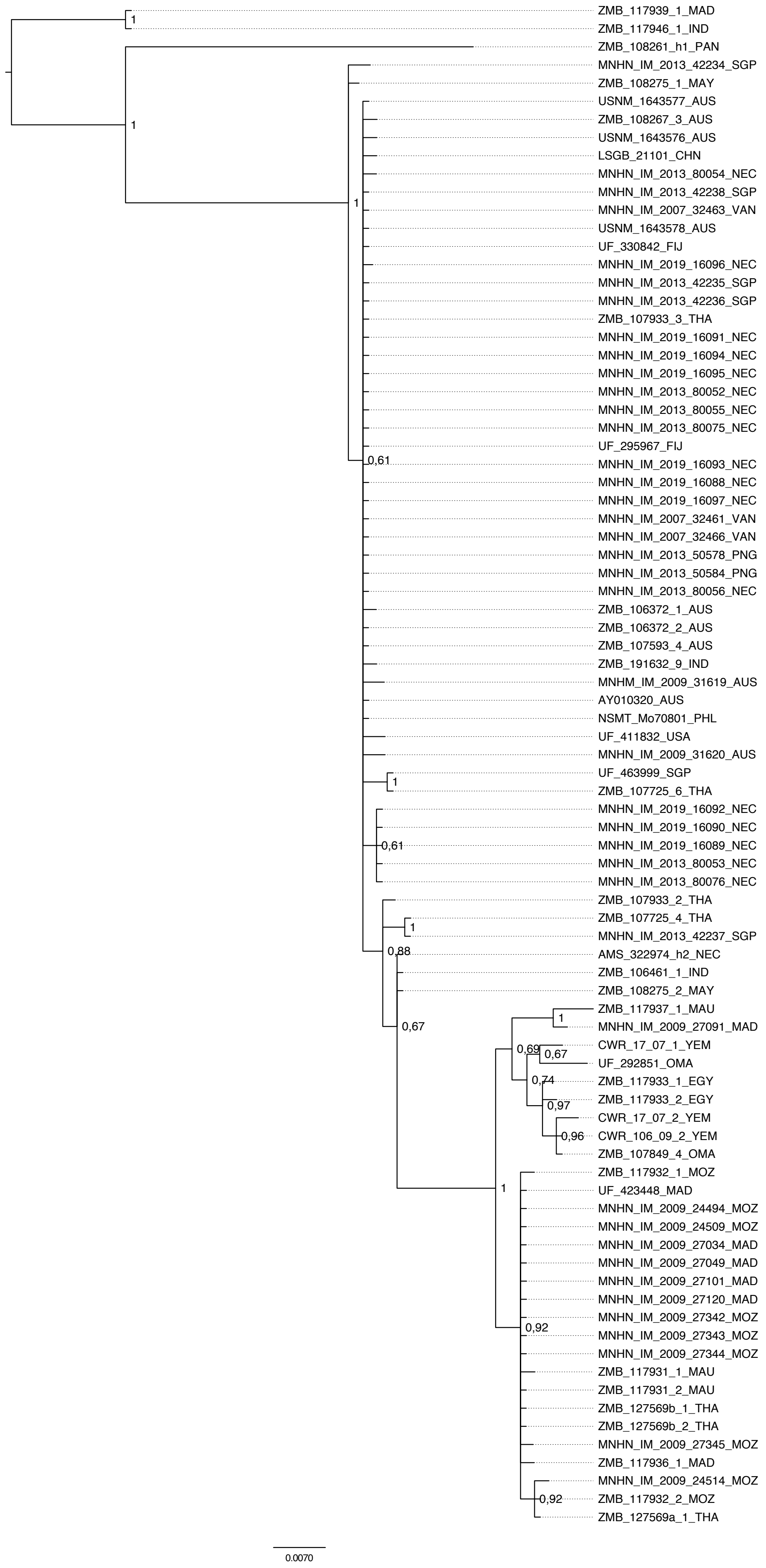

Figure S5. Bayesian tree based on the 16S rRNA dataset.

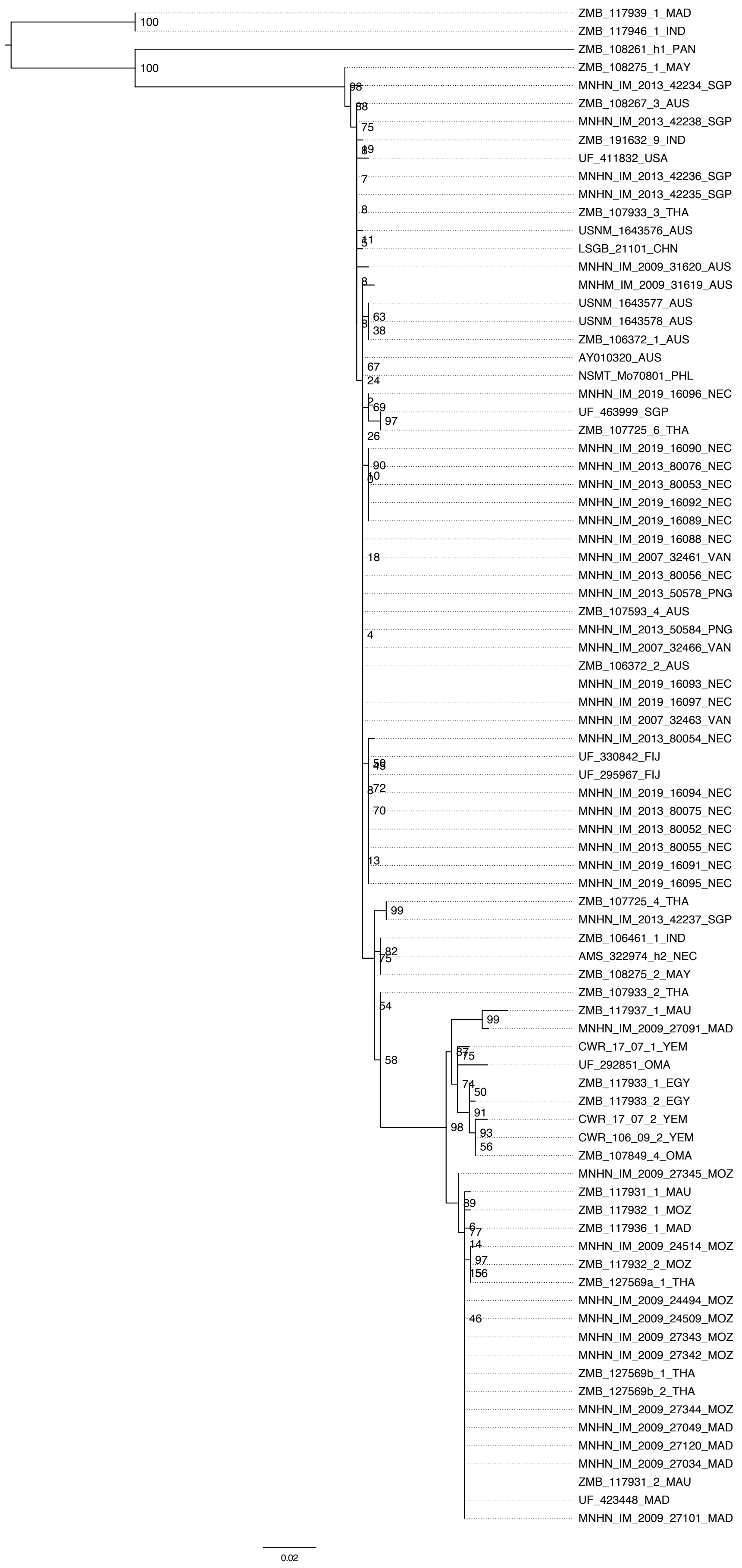

Figure S6. Maximum Likelihood tree based on the 16S rRNA dataset.
